# Are Vitamin D Levels Related to Sarcopenia in Children with Inflammatory Bowel Disease?

**DOI:** 10.3390/jcm14051548

**Published:** 2025-02-25

**Authors:** Sevim Çakar, Gülin Eren, Cahit Barış Erdur, Mehmet Önder, Şafak Pelek, Duygu Demirtaş, Özlem Bekem, Çiğdem Ömür Ecevit

**Affiliations:** 1 Department of Pediatrics, Division of Pediatric Gastroenterology, Faculty of Medicine, Dokuz Eylül University, Izmir 35330, Turkey; 2 Department of Pediatrics, Division of Pediatric Gastroenterology, University of Health Sciences Dr. Behçet Uz Children’s Hospital, Izmir 35210, Turkey

**Keywords:** sarcopenia, pediatrics, vitamin D, psoas muscle area, magnetic resonance

## Abstract

**Background:** Data on the impacts of vitamin D deficiency on sarcopenia in pediatric patients with inflammatory bowel disease (IBD), including Crohn’s disease (CD) and ulcerative colitis (UC), are lacking. We aimed to investigate the relationships between vitamin D levels and sarcopenia in patients with newly diagnosed IBD. **Methods:** A cross-sectional, retrospective study was conducted in a tertiary care children’s hospital. Pediatric IBD patients who underwent magnetic resonance (MR) enteroclysis at the time of initial diagnosis were included. Total psoas muscle area (tPMA) at the L4/L5 intervertebral level was demonstrated on MR by scanning the right and left psoas muscle areas. Sarcopenia was defined as a measurement under the 10th percentile according to MR-derived reference values of tPMA percentile charts for healthy children aged 1–18 years. Vitamin D insufficiency was defined as a serum 25-OH-D level below 30 ng/mL and deficiency as that below 20 ng/mL. Collected data from demographic evaluation, clinic, and laboratory tests were statistically assessed. **Results:** According to the MR-derived reference values of tPMA, 85% (*n* = 33) of UC and 81% (*n* = 21) of CD patients had sarcopenia. The severe vitamin D deficiency ratio was 35.9% (*n* = 14) in UC and 38.5% (*n* = 10) in CD. We found that vitamin D levels were similar in patients with UC and CD, while they were significantly lower in the group below the 3rd percentile of tPMA (*n* = 41, median 9.8) than in the group between the 3rd and 10th percentiles (*n* = 13, median 16.9; *p* = 0.038). **Conclusions:** Formulating strategies to recognize and prevent sarcopenia, including the prevention and—if necessary—the treatment of vitamin D deficiency, could bring multi-faceted benefits.

## 1. Introduction

Ulcerative colitis (UC) and Crohn’s disease (CD), two disorders defined as inflammatory bowel disease (IBD), are chronic diseases that are being diagnosed at an increasingly younger age [1]. Besides characteristic gastrointestinal symptoms, musculoskeletal alterations can manifest as an extraintestinal finding, particularly in cases of CD or as a complication of IBD. Sarcopenia is one of such musculoskeletal findings first defined in older people as a loss of strength and muscle mass, and it is strongly associated with vulnerability to medical stressors and adverse health outcomes [1]. The mechanisms underlying pediatric sarcopenia are not well understood but may be related to the hypermetabolic state, a reduced dietary intake and physical inactivity, an imbalance of gut microbiota, and inflammatory disorders [2]. These risk factors for sarcopenia are part of the pathogenesis of IBD, and, therefore, this condition should be closely evaluated in all IBD patients in both the long and short term. The association between deficient vitamin D and reduced weight-bearing activity may contribute to the development of sarcopenia [2]. Vitamin D deficiency, if diagnosed, can be a modifiable risk factor to prevent the progress of sarcopenia in IBD patients. However, there is still a gap in knowledge regarding the impact of vitamin D deficiency on sarcopenia in pediatric IBD. Sedentary and unhealthy lifestyles, the urban environment, reduced physical stimulation, abdominal obesity, an inadequate protein intake, and an unhealthy diet are considered independent risk factors for sarcopenia [2]. Many etiologies of sarcopenia other than vitamin D deficiency and IBD have been identified in young people. The limitations inherent within the extant published studies are as follows: first, the evaluation of these factors has not been conducted simultaneously; second, the number of patients in studies conducted in pediatric IBD is typically limited.

Considering the pediatric population, the global prevalence of sarcopenia is difficult to estimate; however, it is now seen as a serious problem in the context of pediatric IBD [1,2]. Even though the proportion of young patients with sarcopenia is comparatively lower than the proportion of elderly patients, the greater duration of sarcopenia in youth may ultimately result in a higher incidence of morbidities. In one of the small number of systemic reviews, a lean mass deficit was reported in 47.7% of UC and 93.6% of CD patients of pediatric age [3]. It is crucial to measure muscle mass to diagnose sarcopenia in IBD. However, body mass index (BMI)—commonly used as a nutritional status assessment tool—is not accurate enough to estimate body composition. Muscle mass can be measured using many alternative radiological methods, including ultrasound (US), dual-energy x-ray absorptiometry (DXA), computed tomography (CT), quantitative peripheral CT, and magnetic resonance (MR) [4,5,6,7]. Imaging techniques (CT and MR) are the gold standard for quantifying muscle mass in adults. MR is radiation-free and more expensive than CT; however, in children with IBD, imaging is often performed as a routine part of the evaluation, assessing muscle mass without possible radiation exposure or additional cost [4]. Sex- and age-specific percentile curves of psoas muscle area (PMA) for healthy children obtained from CT scans have recently been published [7,8,9]. Moreover, a recent study has presented MR-derived reference values for PMA in percentile charts for children aged 1 to 18 [10].

Vitamin D is a critical nutrient for bone health, immune modulation, and muscle function [11]. It plays a crucial role in muscle strength by supporting muscle fiber health and reducing inflammation. However, malabsorption, limited dietary intake, reduced sunlight exposure, and chronic inflammation contribute to vitamin D deficiency [12]. In the recent guidelines published by the Endocrine Society, monitoring the 25(OH)D level in patients with malabsorption (e.g., gastrointestinal surgery and inflammatory bowel disease) and enhanced vitamin D catabolism (e.g., due to medications) at the time of diagnosis and during the treatment period is recommended [13]. Furthermore, the special Porto group for IBD of the European Society of Pediatric Gastroenterology, Hepatology, and Nutrition (ESPGHAN) prepared a position paper that includes recommendations for the screening of micronutrient deficiencies in the context of IBD [14]. Moreover, recent evidence has suggested that low vitamin D levels may exacerbate bone density problems and increase the risk of sarcopenia not only in elderly IBD patients but also in children [4,15,16,17]. The role of vitamin D in the development of sarcopenia is not yet fully understood. Specifically, the potential independent role of vitamin D level as a contributing factor, as well as its potential interactive relationships with other factors (e.g., physical activity), remains to be clarified. Furthermore, the complex interplay between vitamin D and muscle function warrants further investigation to fully comprehend the underlying mechanisms.

To the best of our knowledge, this is the first study to use MR-derived sex and age percentiles of TPA to define sarcopenia in children with IBD. Our aim was to investigate the prevalence of sarcopenia in connection with vitamin D levels in newly diagnosed IBD patients. We hypothesized that patients with IBD who had sarcopenia would have lower levels of vitamin D.

## 2. Material and Methods

### 2.1. Participants

A cross-sectional, retrospective study was carried out at a reference children’s hospital between January 2019 and January 2024 and included children younger than 18 years old at the time of diagnosis and followed up for at least one year. The diagnosis of IBD was made based on the revised Porto criteria issued by ESPGHAN for IBD in children and adolescents [18]. Pediatric IBD patients who underwent MR enteroclysis at the initial diagnosis (before taking any steroid or immunosuppressant therapy) were included. MR images with poor quality were not included in the study. In this retrospective study, in order to minimize bias, patient image records were obtained in such a way that the patient’s name was not visible and measurements could be accurately made. Height, weight, and BMI z-scores were calculated according to the 2006 National Growth Charts published by Neyzi et al. (2006) [19]. Patients with professional sports backgrounds, missing data, any chronic or metabolic disease, or anatomic pathology were excluded.

### 2.2. Study Design

The anthropometric measurements, demographic characteristics, laboratory clinical data, disease activity, and all other medical records of children with IBD were obtained retrospectively. The disease activity (mild, moderate, severe) was evaluated using the Pediatric Ulcerative Colitis Activity Index (PUCAI; where PUCAI < 10 indicates remission, 10–34 mild, 35–64 moderate, and >65 severe disease activity) or the Pediatric Crohn’s Disease Activity Index (PCDAI; where PCDAI < 10 indicates remission, 11–30 mild, 31–39 moderate, and >40 severe disease activity) [20,21]. Laboratory and MR data were collected at the time of diagnosis, before corticosteroid or other immunosuppressive treatments.

### 2.3. Muscle Mass Measurement

The pediatric gastroenterologist, blinded to the names and clinical data of the children, measured each PMA at the L4/L5 vertebral levels identified on MR by scanning the right and left psoas regions. To merge the two muscles, a freehand region of interest (ROI) was used via the GE Healthcare PACS. This was considered as the tPMA record. Sarcopenia was defined as measuring below the 10th percentile according to MR-derived reference values from tPMA percentile charts for healthy children aged 1–18 years [10]. Z-scores of tPMA according to the pediatric CT-derived references published by Lurz et al. (2020) were also used for evaluation of the measurements [7].

### 2.4. Laboratory Data

Laboratory tests included serum ferritin, 25(OH)-vitamin D, albumin, C-reactive protein (CRP), and erythrocyte sedimentation rate (ESR). Vitamin D insufficiency was described as a serum 25-OH-D level below 30 ng/mL, and deficiency was considered to be below 20 ng/mL. Liquid chromatography–mass spectrometry (LC-MS) was used to measure vitamin D levels.

### 2.5. Statistical Analysis

The Statistical Package for the Social Sciences (SPSS) 22.0 (IBM Corp, Armonk, NY, USA) was used for all statistical analyses. A *p*-value under 0.05 was considered to indicate a statistically significant result. The Shapiro–Wilk test was used to evaluate the distribution of variables. The results of descriptive analyses are presented as the mean and standard deviation (SD) for normally distributed variables, the median and interquartile range (IQR) for non-normally distributed variables, and percentages for categorical variables. In the case of normally distributed parameters, Student’s *t*-test was used for group comparisons. In cases where the data were not normally distributed, the Mann–Whitney U-test was used to compare the groups. Comparisons of categorical data were made using the chi-square test and Fisher’s exact test when the assumptions did not hold due to low expected cell counts. Pearson or Spearman correlation tests were used to evaluate the correlation coefficients and their significance.

## 3. Results

A total of 65 newly diagnosed IBD patients were enrolled, including 39 (60.0%) UC patients and 26 (40.0%) CD patients. The patients’ demographic, laboratory, anthropometric, and tPMA Z-score data are presented in Table 1.

In 85% (*n* = 22) of patients diagnosed with CD and 82% (*n* = 32) of patients diagnosed with UC, tPMA Z scores were below −1 SD based on CT images. According to MR-derived reference values of tPMA, 85% (*n* = 33) of UC and 81% (*n* = 21) of CD patients had tPMA under the 10th percentile.

The tPMA Z-score medians of mild (*n* = 8), moderate (*n* = 15), and severe (*n* = 12) disease activity groups for UC patients were −1.26 (−3.03 to −0.49), −2.54 (−4.8 to −0.5), and −1.60 (−3.24 to −1.01), respectively. A significantly lower Z-score was found in the pairwise comparison of moderately active compared to severe UC (*p* = 0.047). The tPMA Z-score medians of mild (*n* = 9), moderate (*n* = 6), and severe (*n* = 11) disease activity groups of CD patients were −2.34 (−4.09 to −0.98), −1.37 (−3.36 to 3.26), and −2.45 (−4.01 to −1.34), respectively. The Z-score of tPMA did not statistically differ between the groups, according to CD activity at diagnosis (*p* = 0.114). The ratio of perianal disease did not statistically differ between the sarcopenic (5/54) and non-sarcopenic (2/11) groups (*p* = 0.426).

Demographic and laboratory data of patients with and without sarcopenia are presented in Table 2.

The percentages of all IBD patients with vitamin D levels < 10 ng/mL, between 10 and 20 ng/mL, and >20 ng/mL were 37% (*n* = 24), 46% (*n* = 30), and 17% (*n* = 11), respectively. A total of 54 (83%) patients had vitamin D deficiency. Compared with UC, patients with CD did not have significantly different vitamin D levels (*p* = 0.857) or vitamin D deficiency ratio (*p* = 0.957). The ratio of severe vitamin D deficiency was 35.9% (*n* = 14) in UC and 38.5% (*n* = 10) in CD.

The anthropometric data of patients with IBD showed no correlation between the vitamin D levels and Z-score of tPMA in CD and UC (*p* = 0.382 and *p* = 0.079, respectively). No correlation was found between age and vitamin D levels (*p* = 0.697), while a weak correlation was found between albumin and vitamin D levels (r = 0.283, *p* = 0.024).

Vitamin D levels were statistically lower in the under three-percentile tPMA group (*n* = 41), with a median of 9.8 (3.0–34.2), than in the group with a tPMA percentile between 3 and 10 (*n* = 13), with a median of 16.9 (8.1–31.0; *p* = 0.038); however, no difference was observed with the non-sarcopenic patients (*n* = 11), with a median of 16.3 (6.5–26.1; Table 3).

## 4. Discussion

Muscle strength, physical performance, and muscle mass, as measured via DXA, CT, or MR, are diagnostic tools for sarcopenia. Even though all of these modalities are utilized in pediatrics, it is challenging to standardize the criteria for sarcopenia in children. This is due to a lack of validation data, consensus about the optimal cut-off values, and a large variation in body sizes. The scope of MR studies is comparatively restricted, with the majority of research utilizing data collected via CT scans [7,8,9]. The measurement of muscle mass in abdominal MR images—which are routinely obtained to facilitate the diagnosis of complications in IBD patients—can serve as a guide for sarcopenia in these patients [22]. MR is a non-ionizing method, and its use in diagnosing sarcopenia is superior to CT in this respect. To the best of our knowledge, this is the first study to apply tPMA percentile curves, determined using MR data, to diagnose sarcopenia in pediatric IBD. It is evident that anthropometric measurements are not a good method for assessing sarcopenia, as illustrated in the results. In a way that supports the data in our study, Atlan et al. did not identify a statistically significant correlation between BMI and psoas area but found a significant correlation between the height Z-score and the psoas area of the patients [23]. Another study has shown that BMI is only moderately correlated with muscle mass [24]. Consequently, the measurement of muscle mass assumes even greater significance in diagnosing sarcopenia [25].

One of the most remarkable findings of our study is the very high rate of sarcopenia (85%) in CD and UC (82%) patients. A study by Mager et al., using skeletal muscle mass, reported lower rates of sarcopenia, with 31% in CD and 15% in UC [15]. Another study evaluating sarcopenia via MR imaging reported rates of 43% in pediatric CD patients, much lower than the ratio that we detected (85%), and the mean tPMA was also markedly higher (with 1.1 SD compared to our −2.3 SD) [22]. A major study of pediatric patients supporting such high rates as observed in our data revealed that 95% of individuals diagnosed with IBD demonstrated a psoas area that was below the 25th percentile [23]. The underlying cause of these discrepancies can be attributed to the utilization of divergent imaging modalities, varied references, and the fact that these studies were conducted in small patient groups. Additionally, our country is among the developing nations, and many children—not only IBD patients—are challenged with undernutrition. The study region has traditional Mediterranean dietary habits but, despite this fact, eating habits in the adolescent age group have shifted toward processed fast food-type foods in a manner that is increasing year by year, which may have contributed to the high sarcopenia rate and vitamin D deficiency.

Contrary to the findings of a pediatric study that reported a higher prevalence of sarcopenia in boys, no statistically significant gender disparity was observed in our sarcopenic patients [22]. Supporting our data, Mager et al. also found that sarcopenia was not associated with gender [15].

Interestingly, our study observed that patients with moderate UC activity levels were at higher risk for sarcopenia compared to those with severe UC. This is a result that conflicts with studies supporting an increased risk of sarcopenia with increased inflammation. As patients with severe UC had more severe symptoms and were diagnosed early, it was thought that mild and moderate UC patients, with less muscle loss and a more subtle and gradual onset, were more likely to be diagnosed in the later period. The body’s reaction to inflammation—particularly when there are high levels of inflammatory mediators such as IL-6 and TNF-α—is thought to be linked to the breakdown of muscle and a decrease in muscle growth [5]. Increased disease activity scores are considered an indicator of increased inflammation. Sarcopenia is associated with an increase in disease activity in children with CD [4].

Statistically significant differences were not observed between the sarcopenic and non-sarcopenic groups in terms of serum vitamin D, ferritin, albumin, C-reactive protein (CRP) levels, and erythrocyte sedimentation rate. These parameters are associated with inflammation and, although we expected a significant difference between the groups with and without sarcopenia, no statistical difference was found, which was similar to the study from Mager et al. [15].

Although inflammatory markers were significantly higher in CD, no difference was found between CD and UC patients in terms of sarcopenia. Although the relationship between sarcopenia and inflammation has been shown in many publications, our data prove that many other factors play a role in sarcopenia.

The prevalence of vitamin D deficiency reaches 42–48% in healthy adolescents [26]. A systematic and meta-analysis study has revealed that the prevalence of vitamin D deficiency or insufficiency in children and adolescents with IBD is 44% [27]. The present study established that the rate of vitamin D deficiency in patients diagnosed with IBD was twice as high, with a ratio of 83%. Meanwhile, a serum 25-OH-D concentration below 10 ng/mL indicates severe vitamin D deficiency [14]. Closer to the literature data, the rates of severe vitamin D deficiency in our study were 35.9% in UC and 38.5% in CD patients. In many other pediatric studies in children with IBD, 19–38% of children were diagnosed as vitamin D-deficient [28,29,30]. A study conducted in Israel revealed that 21% of patients presented with vitamin D deficiency at the time of IBD diagnosis, and no significant association was observed with the month, season, or disease type [31]. This finding is of particular significance given that the study region—Turkey—has a similar sunny geographical location and climate as Israel, and the absence of seasonal variations in the data strengthens the study’s conclusions. It is important to note that the study region has high levels of sunlight throughout the year, with no significant seasonal variations in solar exposure. Despite this fact, we observed a very high rate of vitamin D deficiency.

Low vitamin D levels are associated with muscle weakness and atrophy of type II fibers [32]. Serum vitamin D deficiency has been associated with impaired activation of muscle protein synthesis pathways in the skeletal muscle of young adults with CD [33]. In children with newly diagnosed CD, sarcopenia has been found to be associated with vitamin D deficiency [15]. One of the findings supporting our hypothesis is the lower vitamin D levels in the group below the third percentile of tPMA, additionally supported by a previous study in which tPMA was significantly lower in the group with a serum vitamin D level of less than 20 ng/mL [15].

Studies should include further muscle strength and physical function measurements when attempting to identify the relationship between sarcopenia and vitamin D deficiency in IBD patients.

## 5. Study Limitations

The limitations of this study, including its cross-sectional, retrospective design and relatively small sample size, must be acknowledged. Potential confounders such as variations in sun exposure habits, seasonal changes in vitamin D, the degree of reduction in physical activity, and using vitamin D supplementation are other factor that can affect vitamin D levels.

The presence of other extraintestinal manifestations of IBD, genetic factors that may play a role in the etiology, disease location, ethnicity, and other micronutrient status-related factors may also be among the confounding factors.

## 6. Future Perspectives

Sarcopenia in children with IBD has been investigated in a small number of studies, and more data from multi-center and prospective studies are needed. Tools and programs that can easily detect and calculate sarcopenia according to sex and age should be developed. Still, there remains a lack of multi-centric, prospective-designed study data on the relationships between vitamin D deficiency and sarcopenia in pediatric IBD patients.

## 7. Conclusions

Awareness, clear diagnostic criteria, and easily accessible calculation tools are required for pediatric sarcopenia, especially in IBD patients, highlighting the urgent need for effective detection and management strategies. Psoas muscle assessment is a simple, cost-effective, and highly reproducible measurement used in standard MRI studies in children with IBD.

According to the MR-derived reference values of tPMA percentile charts, 85% of patients with UC and 81% of patients with CD had tPMA values below the 10th percentile. Furthermore, 83% of patients had vitamin D deficiency. Moreover, the results indicated that patients with tPMA levels below the third percentile had significantly lower vitamin D levels.

Sarcopenia can be associated with longer hospital stays, increased morbidity and mortality, and health-related complications in children diagnosed with IBD. Consequently, formulating strategies to recognize and prevent sarcopenia, including the prevention and—if necessary—the treatment of vitamin D deficiency, could bring multi-faceted benefits.

## Figures and Tables

**Table 1:** Demographic, anthropometric, and laboratory characteristics of IBD-diagnosed pediatric patients.

	Ulcerative Colitis *n* = 39	Crohn’s Disease *n* = 26	*p*-Value
Age (years), median (min–max)	13 (3–17)	14 (7–17)	0.200
Gender, *n* (%)			0.222
Boys	24 (61.5)	12 (46.2)	
Girls	15 (38.5)	14 (53.8)	
Z-score of BMI median (min–max)	−1.4 (−4.9–2.1)	−1.5 (−6.4–3.2)	0.907
Z-score of weight median (min–max)	−1.2 (−1.3–2.7)	−1.9 (−5–3.5)	0.153
Z-score of height median (min–max)	−0.2 (−2.1–2.1)	−0.9 (−3.6–1.4)	0.027
Z-score of tPMA ^a^ median (min–max)	−2.2 (−4.1–1.0)	−2.3 (−4.1–3.4)	0.388
Serum vitamin D (ng/mL) median (min–max)	13 (5–34)	16 (3–26)	0.857
Serum ferritin (g/dL) median (min–max)	15 (1–316)	86 (7–368)	<0.001
Serum albumin (g/dL) mean ± SD	3.78 ± 0.64	3.43 ± 0.58	0.039
C-reactive protein (mg/L) median (min–max)	1.2 (0.1–13)	5.2 (0.7–16)	<0.001
ESR (mm/h) mean ± SD	46.7 ± 20.4	59.9 ± 18.5	0.015

tPMA, total psoas muscle area; SD, standard deviation; BMI, body mass index., ESR, erythrocyte sedimentation rate. ^a^ According to the calculator providing gender- and age-specific z-scores for tPMA at intervertebral lumbar 4–5 levels in children between the ages of 1 and 16, using reference range charts created after a review of CT scans obtained from healthy children (Lurz et al., 2020) [7].

**Table 2:** Demographic, anthropometric, and laboratory characteristics of IBD-diagnosed patients with and without sarcopenia.

	Sarcopenic Patients *n* = 54	Non-Sarcopenic Patients *n* = 11	*p*-Value
Age (years) median (min–max)	14 (3–17)	12 (7–17)	0.685
Gender, *n* (%)			0.546
Boys	29 (53.7)	7 (63.6)	
Girls	25 (46.3)	4 (36.4)	
Diagnosis UC *n* (%) CD *n* (%)	33 (61.1) 21 (38.9)	6 (54.5) 5 (45.5)	0.685
Z score of BMI mean ± SD	−1.8 ± 1.6	−0.1 ± 1.9	0.005
Z score of weight median (min–max)	−1.8 (−5.0–2.7)	−0.4 (−1.8–3.5)	0.003
Z score of height mean ± SD	−0.5 ± 1.3	−0.4 ± 0.8	0.030
Z score of tPMA ^a^ median (min–max)	−2.5 (−4.1–−0.6)	−0.5 (−1.3–3.4)	<0.001
Serum vitamin D (ng/mL) mean ± SD	14.4 ± 6.9	15.2 ± 6.0	0.416
Serum ferritin (g/dL) median (min–max)	21.3 (1–316)	21.4 (3–368)	0.849
Serum albumin (g/dL) mean ± SD	3.6 ± 0.7	3.7 ± 0.5	0.938
C-reactive protein (mg/L) median (min–max)	2.8 (0.1–16.0)	2.8 (0.7–7.0)	0.850
ESR (mm/h) median (min–max)			
	48 (13–97)	46 (28–79)	0.392

tPMA, total psoas muscle area; SD, standard deviation; BMI, body mass index, ESR, erythrocyte sedimentation rate. ^a^ According to the calculator providing gender- and age-specific z-scores for tPMA at intervertebral lumbar 4–5 levels in children between the ages of 1 and 16, using reference range charts created after a review of CT scans obtained from healthy children (Lurz et al., 2020) [7].

**Table 3:** tPMA percentiles according to vitamin D levels.

	*n* and % of Patients with tPMA < 3 Percentile ^α^	*n* and % of Patients with tPMA 3–10 Percentile ^α^	*n* and % of Patients with tPMA > 10 Percentile ^α^	*p*-Value
* Serum vitamin D below 10 ng/mL	21 (51.2)	1 (7.7)	2 (18.2)	^§^ 0.035
^+^ Serum vitamin D between 10 and 20 ng/mL	15 (36.6)	9 (69.2)	6 (54.5)	
Serum vitamin D above 20 ng/mL	5 (12.2)	3 (23.1)	3 (27.3)	

^α^ According to MR-derived reference values of tPMA percentile charts for boys and girls aged from 1–18 [10]. ^§^ The significant difference derives from two groups (* and ^+^) in post hoc analyses.

## Data Availability

The corresponding author will share the data upon request due to legal and ethical reasons.

## References

[1] Shaw K.A., Bertha M., Hofmekler T., Chopra P., Vatanen T., Srivatsa A., Prince J., Kumar A., Sauer C., Zwick M.E. (2016). Dysbiosis, inflammation, and response to treatment: A longitudinal study of pediatric subjects with newly diagnosed inflammatory bowel disease. Genome Med..

[2] Jung H.N., Jung C.H., Hwang Y.C. (2023). Sarcopenia in youth. Metabolism.

[3] Thangarajah D., Hyde M.J., Konteti V.K.S., Santhakumaran S., Frost G., Fell J.M.E. (2015). Systematic review: Body composition in children with inflammatory bowel disease. Aliment. Pharmacol. Ther..

[4] Ooi P.H., Thompson-Hodgetts S., Pritchard-Wiart L., Gilmour S.M., Mager D.R. (2020). Pediatric Sarcopenia: A Paradigm in the Overall Definition of Malnutrition in Children?. J. Parenter. Enter. Nutr..

[5] Celentano V., Kamil-Mustafa L., Beable R., Ball C., Flashman K.G., Jennings Z., Leary D.P.O., Higginson A., Luxton S. (2021). Preoperative assessment of skeletal muscle mass during magnetic resonance enterography in patients with Crohn’s disease. Updates Surg..

[6] Koltzenburg M., Yousry T. (2007). Magnetic resonance imaging of skeletal muscle. Curr. Opin. Neurol..

[7] Lurz E., Patel H., Lebovic G., Quammie C., Woolfson J.P., Perez M., Ricciuto A., Wales P.W., Kamath B.M., Chavhan G.B. (2020). Pediatric reference values for total psoas muscle area. J. Cachexia Sarcopenia Muscle.

[8] Ritz A., Lurz E., Berger M. (2022). Sarcopenia in Children with Solid Organ Tumors: An Instrumental Era. Cells.

[9] Romano A., Triarico S., Rinninella E., Natale L., Brizi M.G., Cintoni M., Raoul P., Maurizi P., Attinà G., Mastrangelo S. (2022). Clinical impact of Nutritional Status and Sarcopenia in Pediatric patients with bone and soft tissue sarcomas: A pilot retrospective study (SarcoPed). Nutrients.

[10] Marunowski K., Świętoń D., Bzyl W., Grzywińska M., Bandosz P., Khrichenko D., Piskunowicz M. (2022). Reference values for MRI-derived psoas and paraspinal muscles and macroscopic fat infiltrations in paraspinal muscles in children. J. Cachexia Sarcopenia Muscle.

[11] Del Pinto R., Pietropaoli D., Chandar A.K., Ferri C., Cominelli F. (2015). Association between inflammatory bowel disease and vitamin D deficiency: A systematic review and meta-analysis. Inflamm. Bowel Dis..

[12] Holick M.F., Binkley N.C., Bischoff-Ferrari H.A., Gordon C.M., Hanley D.A., Heaney R.P., Murad M.H., Weaver C.M. (2011). Evaluation, treatment, and prevention of vitamin D deficiency: An Endocrine Society clinical practice guideline. J. Clin. Endocrinol. Metab..

[13] Demay M.B., Pittas A.G., Bikle D.D., Diab D.L., Kiely M.E., Lazaretti-Castro M., Lips P., Mitchell D.M., Murad M.H., Powers S. (2024). Vitamin D for the prevention of disease: An Endocrine Society clinical practice guideline. J. Clin. Endocrinol. Metab..

[14] Miele E., Shamir R., Aloi M., Assa A., Braegger C., Bronsky J., de Ridder L., Escher J.C., Hojsak I., Kolaček S. (2018). Nutrition in pediatric inflammatory bowel disease: A position paper on behalf of the Porto Inflammatory Bowel Disease Group of the European Society of Pediatric Gastroenterology, Hepatology and Nutrition. J. Pediatr. Gastroenterol. Nutr..

[15] Mager D.R., Carroll M.W., Wine E., Siminoski K., MacDonald K., Kluthe C.L., Medvedev P., Chen M., Wu J., Turner J.M. (2018). Vitamin D status and risk for sarcopenia in youth with inflammatory bowel diseases. Eur. J. Clin. Nutr..

[16] Çakar S., Eren G. (2023). Diagnosis of sarcopenia with magnetic resonance imaging of newly diagnosed pediatric inflammatory bowel disease. Ann. Clin. Anal. Med..

[17] Bae E.J., Kim Y.H. (2017). Factors affecting sarcopenia in Korean adults by age groups. Osong Public Health Res. Perspect..

[18] Levine A., Koletzko S., Turner D., Escher J.C., Cucchiara S., de Ridder L., Kolho K., Veres G., Russell R.K., Paerregaard A. (2014). ESPGHAN revised Porto criteria for the diagnosis of inflammatory bowel disease in children and adolescents. J. Pediatr. Gastroenterol. Nutr..

[19] Neyzi O., Furman A., Bundak R., Gunoz H., Darendeliler F., Bas F. (2006). Growth references for Turkish children aged 6 to 18 years. Acta Pediatr..

[20] Turner D., Otley A.R., Mack D., Hyams J., De Bruijne J., Uusoue K., Walters T.D., Zachos M., Mamula P., Beaton D.E. (2007). Development, validation, and evaluation of a pediatric ulcerative colitis activity index: A prospective multicenter study. Gastroenterology.

[21] Turner D., Levine A., Escher J.C., Griffiths A.M., Russell R.K., Dignass A., Dias J.A., Bronsky J., Braegger C.P., Cucchiara S. (2012). Management of pediatric ulcerative colitis: Joint ECCO and ESPGHAN evidence-based consensus guidelines. J. Pediatr. Gastroenterol. Nutr..

[22] Blagec P., Sara S., Tripalo Batoš A., Trivić Mažuranić I., Močić Pavić A., Mišak Z., Hojsak I. (2023). Magnetic Resonance Imaging Can Be Used to Assess Sarcopenia in Children with Newly Diagnosed Crohn’s Disease. Nutrients.

[23] Atlan L., Cohen S., Shiran S., Sira L., Pratt L.T., Yerushalmy-Feler A. (2021). Sarcopenia is a predictor for adverse clinical outcome in pediatric inflammatory bowel disease. J. Pediatr. Gastroenterol. Nutr..

[24] Metzger G.A., Sebastião Y.V., Carsel A.C., Nishimura L., Fisher J.G., Deans K.J., Minneci P.C. (2021). Establishing reference values for lean muscle mass in the pediatric patient. J. Pediatr. Gastroenterol. Nutr..

[25] Scaldaferri F., Pizzoferrato M., Lopetuso L.R., Musca T., Ingravalle F., Sicignano L.L., Mentella M., Miggiano G., Mele M.C., Gaetani E. (2017). Nutrition and IBD: Malnutrition and/or Sarcopenia? A Practical Guide. Gastroenterol. Res. Pract..

[26] Gordon C.M., DePeter K.C., Feldman H.A., Grace E., Emans S.J. (2004). Prevalence of vitamin D deficiency among healthy adolescents. Arch. Pediatr. Adolesc. Med..

[27] Fatahi S., Alyahyawi N., Albadawi N., Mardali F., Dara N., Sohouli M.H., Prabahar K., Rohani P., Koushki N., Sayyari A. (2023). The association between vitamin D status and inflammatory bowel disease among children and adolescents: A systematic review and meta-analysis. Front. Nutr..

[28] Levin A.D., Wadhera V., Leach S.T., Woodhead H.J., Lemberg D.A., Czarina Mendoza-Cruz A., Day A.S. (2021). Vitamin D deficiency in children with inflammatory bowel disease. Dig. Dis. Sci..

[29] Pappa H.M., Gordon C.M., Saslowsky T.M., Zholudev A., Horr B., Shih M.C., Grand R.J. (2006). Vitamin D status in children and young adults with inflammatory bowel disease. Pediatrics.

[30] Sentongo T.A., Semaeo E.J., Stettler N., Piccoli D.A., Stallings V.A., Zemel B.S. (2002). Vitamin D status in children, adolescents, and young adults with Crohn disease. Am. J. Clin. Nutr..

[31] Brandvayman Y., Rinawi F., Shamir R., Assa A. (2007). Associations of seasonal patterns and vitamin D levels with onset and flares of pediatric inflammatory bowel disease. Minerva Pediatr..

[32] Roth S.M., Zmuda J.M., Cauley J.A., Shea P.R., Ferrell R.E. (2004). Vitamin D receptor genotype is associated with fat-free mass and sarcopenia in elderly men. J. Gerontol. A Biol. Sci. Med. Sci..

[33] van Langenberg D.R., Della Gatta P., Hill B., Zacharewicz E., Gibson P.R., Russell A.P. (2014). Delving into disability in Crohn’s disease: Dysregulation of molecular pathways may explain skeletal muscle loss in Crohn’s disease. J. Crohns Colitis.

